# Microsaccades are sensitive to word structure: A novel approach to study language processing

**DOI:** 10.1038/s41598-017-04391-4

**Published:** 2017-06-21

**Authors:** Maya Yablonski, Uri Polat, Yoram S. Bonneh, Michal Ben-Shachar

**Affiliations:** 1 The Gonda Multidisciplinary Brain Research Center, Bar Ilan University, Ramat-Gan, Israel; 2 School of Optometry and Vision Science, Mina & Everard Goodman Faculty of Life Sciences, Bar Ilan University, Ramat-Gan, Israel; 3 Department of English Literature and Linguistics, Bar Ilan University, Ramat-Gan, Israel

## Abstract

Microsaccades are miniature eye movements that occur involuntarily during fixation. They are typically inhibited following stimulus onset and are released from inhibition about 300 ms post-stimulus. Microsaccade-inhibition is modulated by low level features of visual stimuli, but it is currently unknown whether they are sensitive to higher level, abstract linguistic properties. To address this question, we measured the timing of microsaccades while subjects were presented with written Hebrew words and pronounceable nonwords (pseudowords). We manipulated the underlying structure of pseudowords such that half of them contained real roots while the other half contained invented roots. Importantly, orthographic similarity to real words was equated between the two conditions. Microsaccade onset was significantly slower following real-root compared to invented-root stimuli. Similar results were obtained when considering post-stimulus delay of eye blinks. Moreover, microsaccade-delay was positively and significantly correlated with measures of real-word similarity. These findings demonstrate, for the first time, sensitivity of microsaccades to linguistic structure. Because microsaccades are involuntary and can be measured in the absence of overt response, our results provide initial evidence that they can be used as a novel physiological measure in the study of language processes in healthy and clinical populations.

## Introduction

Microsaccades are miniature rapid eye movements that occur involuntarily during fixation. While their exact contribution to vision is still debated^1, 2^, their direction and timing have been linked to anticipation, surprise, and attentional shifts^3–8^. Microsaccades are typically inhibited for approximately 300 ms post stimulus onset^5, 9^. Perceptual manipulations (e.g., of stimulus contrast or spatial frequency) result in delayed release from microsaccade-inhibition as processing time increases^10^. Accumulating evidence from recent years suggests that the rate and latency of microsaccades can serve as measures of covert attention^11–13^. However, this was mainly tested in perceptual tasks involving detection of low-level features. To date, the applicability of microsaccades as a sensitive online measure in psycholinguistics has not yet been examined.

Few studies have tested whether microsaccades are sensitive to higher-level cognitive processes. Two studies reported a decrease in the rate of microsaccades for demanding arithmetic computations compared to easy ones^14, 15^. Yet another study has shown that unpleasant pictures induce a decrease in microsaccade-rate^16^. Taken together, these findings imply that microsaccades may be modulated by both cognitive load and emotional state. However, it remains unclear whether microsaccades are sensitive to the rich information provided by structured linguistic stimuli. The aim of the current study was to assess the sensitivity of microsaccades to linguistic properties, specifically, word structure. To this end, we presented participants with complex Hebrew stimuli, and tested whether the rate and latency of microsaccades are modulated by their structural properties.

Words in any human language have an internal structure. The building blocks of words are known as morphemes. For example, the word “unthinkable” is composed of 3 morphemes, un + think + able. Morphemes provide a natural tool for extending the set of words in a language, by combining sets of familiar units to generate novel combinations (e.g., unlaughable). In Hebrew, morphological structure takes a non-linear form: a triconsonantal root (e.g., S.G.R.) is interleaved within a pattern (e.g., miXYeZet, a nominal pattern where X, Y, Z are place holders for the 3 consonants of the root), resulting in the noun *miSGeRet* (a frame). The pattern adds information about the phonological form of the word (vowels are otherwise unspecified in the standard Hebrew script) and modulates the root meaning to express a specific concept. It has been suggested that rapid morphemic decomposition is an essential component of efficient reading in Hebrew^17^.

**Figure 1:**
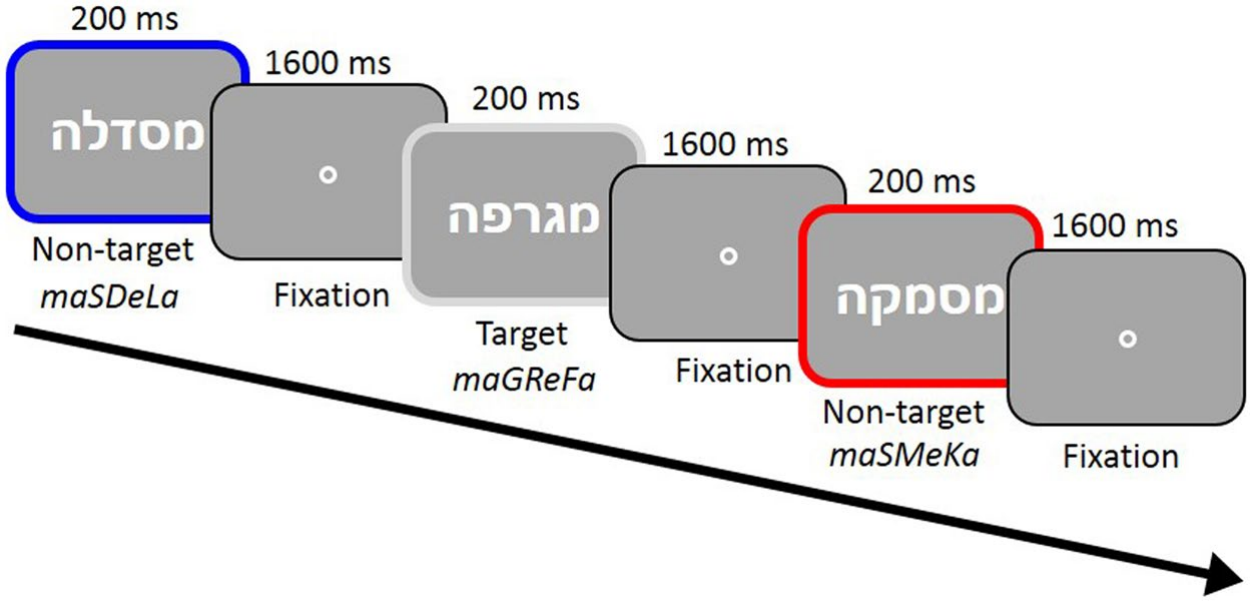
Trial structure and experimental conditions. Stimuli were presented in Hebrew unpointed script (the standard form of presentation for adult readers), in white font on a gray background. Participants performed a word detection task (push a button when the stimulus is an existing word in Hebrew, and do not respond otherwise). Stimulus condition is denoted by frame color: Invented-root pseudowords (blue), Real-root pseudowords (red) and Words (gray) which served as targets in this task. No colors were used in the actual stimuli. The phonetic transcription of each stimulus is presented below it, with the root letters in uppercase. The full list of stimuli may be obtained from the authors.

To test whether microsaccades are sensitive to morphological structure, we adapted a well-known psycholinguistic phenomenon, the “Morpheme Interference Effect” (MIE). This effect is typically demonstrated by comparing two types of stimuli, which are both made-up, pronounceable letter strings (pseudowords). The critical comparison is between pseudowords that contain a non-existing combination of real morphemes (e.g., *shootment*) *vs*. those that contain an invented morpheme (e.g., *shootmant*). By presenting such stimuli in a lexical decision task (word or nonword judgment), it has been shown repeatedly that participants take longer to reject pseudowords made of real morphemes and make many more mistakes on such stimuli, compared to pseudowords containing invented morphemes^18, 19^. This effect cannot be explained by perceptual similarity to real words (because word-similarity is carefully equated between the conditions) and is thus considered behavioral indication that readers extract information about word structure. We recently established that the classical MIE generalizes to nonlinear morphological systems, such as Hebrew^20^. In the current study, we tested whether microsaccades are sensitive to word structure information, by comparing the pattern of microsaccades for pseudowords containing a real root *vs*. pseudowords containing an invented root (see Fig. 1). If microsaccades are indeed sensitive to word structure, we expected a significant delay in microsaccade occurrence following the presentation of pseudowords containing a real root, compared to those containing an invented root.

## Results

### Behavioral task performance

Participants performed the word detection task at high accuracy (96% ± 2.5%; N = 16). We chose this task to avoid task-related motor responses during pseudoword trials, which were the focus of our analysis (see Methods). A paired t-test revealed a significant difference in false alarm levels (i.e., wrong “word” responses to pseudoword stimuli, t(15) = 5.19; p < 0.001), such that more errors were made for Real-root pseudowords (10.5% ± 7.8) compared to Invented-root pseudowords (1.7% ± 1.5; See Supplementary Figure S1 for individual error rates of all subjects). This finding establishes MIE at the behavioral level in the current sample, and generalizes it across tasks from the classically used lexical decision task to the word detection task used here.

### The effect of morphological structure on microsaccade inhibition

**Figure 2:**
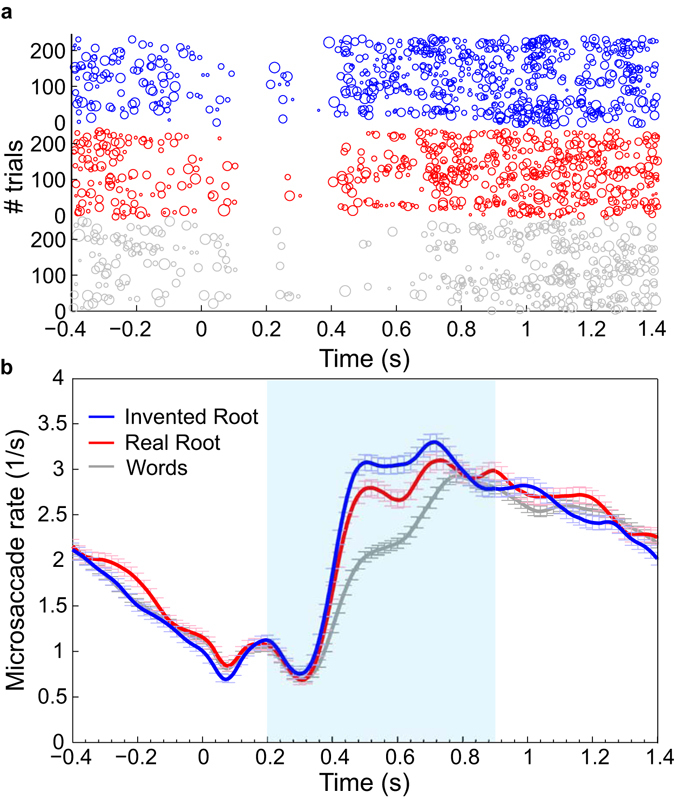
Microsaccade rate modulation. (**a**) Raster plots of microsaccades in a single subject (female, 24y) for Invented-root pseudowords (blue), Real-root pseudowords (red) and Words (gray). Each row represents a single trial. Each circle represents a single microsaccade, with circle diameter proportional to microsaccade magnitude. For visualization, all pseudoword trials (N = 240 trials per condition) but only half of the word trials (N = 240) are displayed. (**b**) Average microsaccade rate modulation curves. Data were averaged, per condition, across all trials and all subjects. Error bars denote standard error of the mean across all trials in each condition. Time zero represents stimulus onset. Shaded blue area illustrates the time window (200–900 ms) used for subsequent analyses.

**Figure 3:**
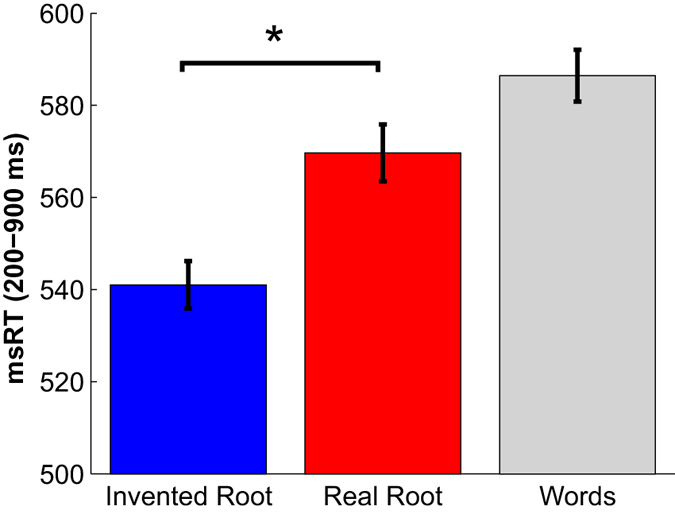
The effect of morphological structure on microsaccade inhibition. msRT is presented for each condition (same color scheme as in Fig. 2). Only trials that included a microsaccade in the specified time window (200–900 ms) were included in the calculation (about 67% of the trials in each condition). Data were normalized (de-meaned) per subject, averaged across subjects (N = 16) and adjusted by adding the group grand-average (see Methods). Error bars denote standard errors across subjects. Microsaccades in Real-root trials were significantly delayed compared to Invented-root trials (nonparametric permutation test). *p < 0.001.

Eye tracking analyses were conducted after excluding false alarm trials (pseudowords wrongly classified as words) to ensure that the results are free from motor artifacts evoked by button presses (exclusion rate ranged across subjects from 1% to 14%, with an average of 6%). We first analyzed the microsaccade-rate modulation, time locked to stimulus onset (Fig. 2). All conditions showed a clear inhibition prior to stimulus onset, presumably reflecting stimulus anticipation based on the fixed SOA. This inhibition was followed by a sharp increase in microsaccade-rate around 300 ms post-stimulus, in accordance with prior measurements using perceptual manipulations (e.g. ref. 10). Critically, release from inhibition was faster and reached a higher peak-rate for Invented-root pseudowords compared to Real-root pseudowords (for statistical analysis see Fig. 3 and accompanying text). This effect suggests that readers extract and process the root while they process written Hebrew stimuli. Importantly, the difference between real and invented pseudowords is unlikely to be explained through orthographic similarity to real words, because this factor was carefully controlled and equated between the conditions (see Methods and Table 1).

**Table 1:** Orthographic properties of pseudoword stimuli equated between the two experimental conditions.

Variable	Real root e.g., (*maSMeKa*)		Invented Root e.g., (*maSDeLa*)		t (478)	p
	M	SD	M	SD		
Length	5.00	0.78	4.98	0.75	0.18	0.86
N	11.79	6.54	12.10	5.81	−0.55	0.58
OLD20	1.32	0.26	1.34	0.25	−0.87	0.38
MLBF	3.47	0.36	3.47	0.37	−0.19	0.85

Length = number of letters; N = number of orthographic neighbors; OLD20 = mean orthographic Levenshtein distance to the 20 closest neighbors; MLBF = mean log bigram frequency.

Rate modulation curves are highly sensitive to the choice of smoothing parameters used to generate them from the raw data. We therefore used these curves mainly for visualization and initial data exploration. To better quantify microsaccade timing, we extracted, for each trial, the microsaccade response time (msRT), which is the latency of the first microsaccade following stimulus onset (see Methods, see also refs 10 and 21). Mean msRT for each condition is shown in Fig. 3. This analysis confirmed that real roots induced a significant delay of 28 ms in microsaccade occurrence for Real-root pseudowords compared to Invented-root pseudowords (p < 0.001, nonparametric permutation test). A parallel analysis limiting the magnitude of microsaccades to 1° yielded similar results (see Supplementary Figs S2–3). These results suggest that microsaccade-inhibition was modulated by the occurrence of the root morpheme within the pseudoword stimuli.

### The effect of morphological structure on blink inhibition

**Figure 4:**
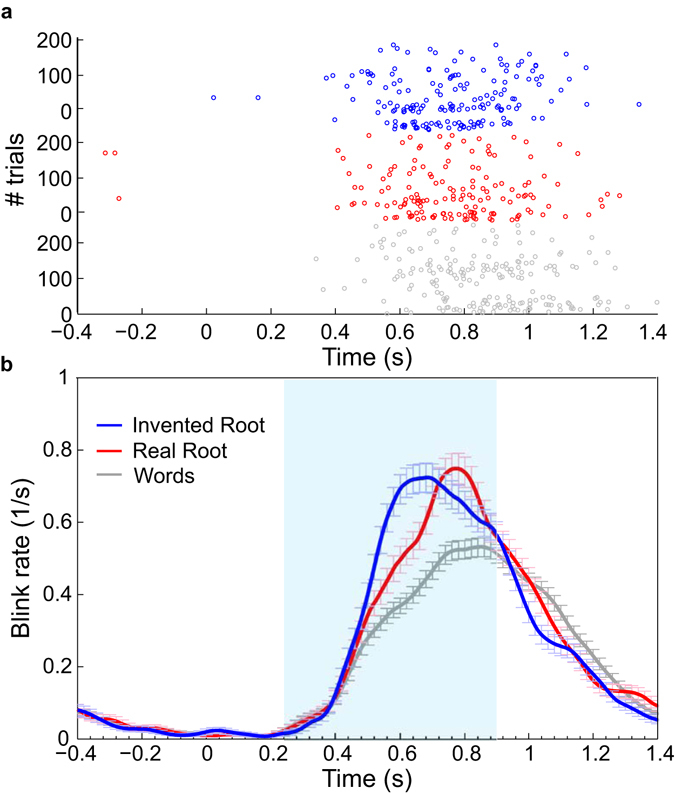
Blink rate modulation. This figure parallels Fig. 2 for blinks. (**a**) Raster plot of blinks of a single subject (female, 24y). Each row represents a single trial, each circle represents a single blink. For visualization, all pseudoword trials (N = 240 trials per condition) but only half of the word trials (N = 240) are displayed. (**b**) Average blink rate modulation. Data were averaged across all trials of all subjects within a condition. Error bars denote standard error of the mean across all trials in each condition. Time zero represents stimulus onset. Shaded blue area illustrates the time window (200–900 ms) used for subsequent analyses.

**Figure 5:**
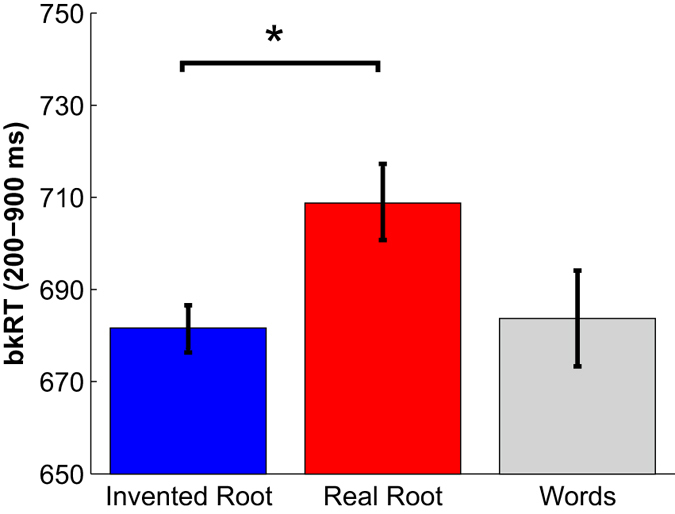
The effect of morphological structure on blink inhibition. Mean blink RT (bkRT; the latency of the first blink following stimulus onset) is averaged across trials and then across subjects, for each condition. N = 12 after excluding 4 subjects due to low blink rate (<10% of trials). Only trials that had a blink in the specified time window (200–900 ms) were included in the calculation (24% of trials in each condition on average). Data were normalized (de-meaned) per subject, averaged across subjects and adjusted by adding the group grand-average (see Methods). Error bars denote standard error across all subjects. As shown, blinks in Real-root trials were significantly delayed by 27 ms on average, compared to Invented-root trials (nonparametric permutation test). *p < 0.001.

**Figure 6:**
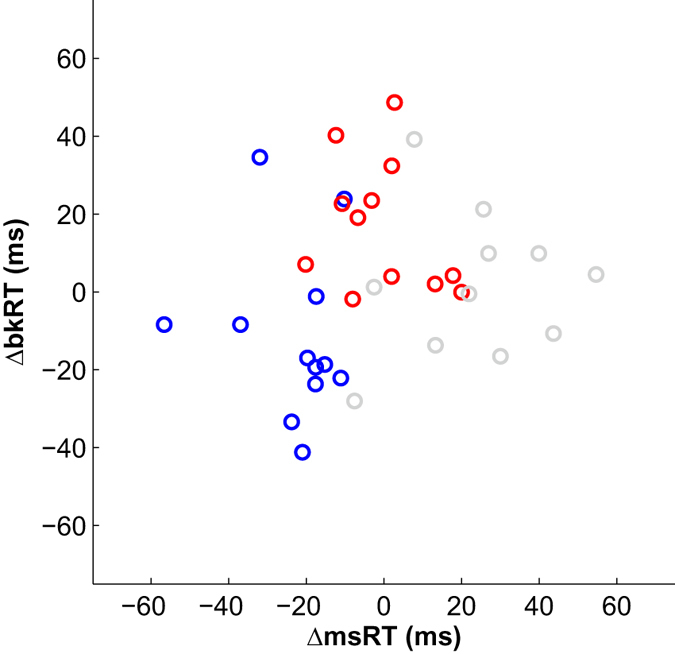
The relationship between msRT and bkRT, across subjects. ∆msRT represents the timing of microsaccades in each condition, after subtracting the average msRT across all conditions. Similarly, ∆bkRT represents the timing of blinks in each condition, after subtracting the average bkRT across all conditions. Each circle represents a single subject’s average ∆msRT (x- axis) and ∆bkRT (y-axis) in a single condition. Thus, for each subject, three dots are displayed, representing Invented-root (blue), Real-root (red) and Words (gray). There is a clear segregation between Real-root pseudowords and Invented-root pseudowords in the timing of both microsaccades and blinks.

Recent findings have shown that eye blink inhibition provides information comparable to microsaccade inhibition in response to visual contrast, suggesting that blinks and microsaccades may share a common mechanism in response to sensory manipulations^21^. We therefore examined the effect of morphemic structure on blinks (Figs 4–5). Four subjects were excluded from this analysis due to extremely low blink rate (<10% of the trials, as in ref. 21). As shown in Fig. 4, Real-root pseudowords incurred a delay in blinks, similar to the one observed for microsaccades. To quantify this effect, we extracted blink response times (bkRT), the timing of the first blink following stimulus onset, and compared the mean bkRT between the two pseudoword conditions. As shown in Fig. 5, bkRT was delayed by 27 ms for Real-root pseudowords compared to Invented-root pseudowords (p < 0.001, nonparametric permutation test), nearly identical to the delay obtained for microsaccades. These findings suggest that blink inhibition is sensitive to morphological structure in Hebrew pseudowords, as is the case for microsaccades. Figure 6 delineates the distribution of these measures (msRT and bkRT) across individual subjects, showing a clear segregation between the Real-root and Invented-root pseudoword conditions.

### Microsaccade inhibition and word similarity

Orthographic similarity to real words is a potential confound in MIE studies: the more a pseudoword resembles a real word, the harder it is to reject, and as a result, the longer the RT to it in a lexical decision task^22^. To ascertain that the delays in microsaccades and blinks are driven by the root morpheme and not by orthographic similarity to existing words, pseudowords in the Real-root and Invented-root conditions were carefully equated on several measures of word-similarity. The variability in word-similarity further allowed us to examine whether microsaccade timing is modulated by word similarity, within each condition. To this end, we calculated correlations between ∆msRT (for each stimulus, the difference between the msRT to that stimulus and the average msRT of the participant), and three measures of word similarity calculated for the same stimulus (see Methods for precise definitions of each measure). This analysis was carried out separately for pseudowords in the Real-root and Invented-root conditions.

**Figure 7:**
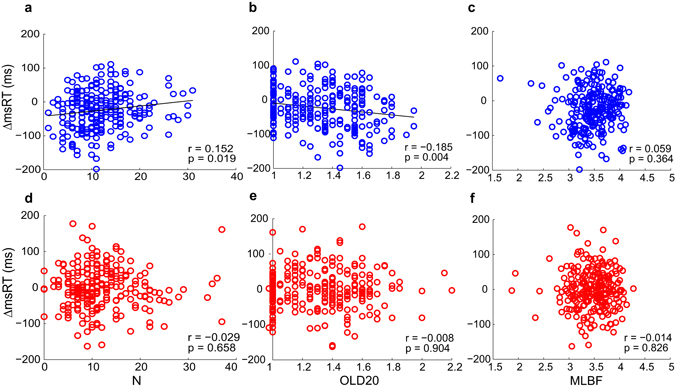
Correlation between word-similarity and microsaccade-timing. (**a**–**c**) Invented-root condition. (**d**–**f**) Real-root condition. In each plot, each datapoint represents the mean ∆msRT per stimulus (N = 240 in each condition) across participants. ∆msRT calculated for each stimulus as explained in Fig. 6, and then averaged per stimulus across participants. In the invented-root condition, significant Pearson’s correlations were found between ∆msRT and Coltheart’s N, and between ∆msRT and OLD20 (panels a,b, black lines represent the best linear fit of the two variables). No significant correlations were found with ∆msRT in the Real-root condition. N = number of orthographic neighbors; OLD20 = mean orthographic Levenshtein distance to the 20 closest neighbors; MLBF = mean log bigram frequency.

This analysis revealed that microsaccade delays to Invented-root pseudowords were indeed significantly associated with word-similarity (Fig. 7a,b). Specifically, ∆msRT to Invented-root pseudowords was negatively correlated with OLD20, the average distance to real-word neighbors^23^ (r = −0.185, p < 0.05, FDR corrected for 6 comparisons, CI_95%_ [−0.3, −0.066]) and positively correlated with Coltheart’s N, the number of real-word neighbors^24^ (r = 0.152, p < 0.05, did not survive FDR correction, CI_95%_ [0.036, 0.253]). These results show that microsaccades are delayed longer for Invented-root pseudowords that resemble real words more. The opposite direction of the correlations with OLD20 and N is expected, because increased OLD20 signifies less word-similarity (larger distance to nearest word), while increased N signifies more word-similarity (more word neighbors). Indeed, N and OLD 20 are typically inversely correlated (in our stimuli: r = −0.736, p < 0.001).

Unlike Invented-root pseudowords, ∆msRT to Real-root pseudowords was not modulated by word similarity (r = −0.029, CI_95%_ [−0.157, 0.138], r = −0.008, CI_95%_ [−0.123, 0.114], and −0.014, CI_95%_ [−0.127, 0.105], n.s., for N, OLD20 and MLBF, respectively; see Fig. 7d–f). Moreover, the correlations reported above for Invented-root pseudowords were significantly stronger than those found with Real-root pseudowords, for both N (z = 1.98, p < 0.05) and OLD20 (z = −1.95, p < 0.05). Taken together, these results imply that microsaccades are sensitive to several levels of linguistic features. When word similarity is controlled for, microsaccade latency is delayed for Real-root pseudowords, showing a sensitivity to morphological structure. In the absence of a real root, microsaccade latency to Invented-root pseudowords is modulated by orthographic similarity to real words, revealing a sensitivity to orthographic features.

## Discussion

Our results reveal that microsaccade timing is modulated by word structure and by similarity to real words. Real roots embedded within a pseudoword evoke delayed release from microsaccade inhibition compared to invented roots, indicating that the root is accessed during visual processing of written stimuli. In pseudowords devoid of a real root, increased orthographic similarity to real words causes a delay in microsaccades. These findings indicate, for the first time, that microsaccades are sensitive to two types of linguistic information: morphological and orthographic structure. The time course of the release from inhibition is in accordance with studies of microsaccades in other high-order cognitive processes^15, 16^. This extends the phenomenon of microsaccade inhibition to the psycholinguistic domain and suggests that microsaccades can serve as a tool for studying implicit language processing.

In addition to increased delay in microsaccades, real root morphemes also induced a delay in the timing of eye blinks. Previous studies have established that the rate of spontaneous eye blinks is lower during reading than during rest or conversation^25–27^. However, little attention was assigned to dynamics of eye blinks during online reading and to differences in blink rate elicited by different types of orthographic stimuli. Our findings demonstrate for the first time that blink timing is sensitive to linguistic content. Despite having a lower rate in general, the results for blinks resemble those obtained for microsaccades, in accordance with recent reports of similar inhibition patterns for these two ocular measures^21, 28^. Our findings provide further support for the notion that the inhibition of both microsaccades and blinks reflects an inhibitory mechanism, which suppresses oculomotor activity in the face of cognitive challenge.

The delay induced by real roots in microsaccade and blink timing suggests that readers automatically extract the root morpheme when they process written Hebrew stimuli. This finding adds to prior behavioral evidence^20^ and contributes to one of the fundamental debates in psycholinguistics: Are words perceived as whole entities or are they decomposed into their constituent morphemes quickly and automatically^18, 29–36^. The selective delay of microsaccades and blinks induced by real root morphemes provides additional support for the idea that the root is accessed when pseudowords are presented, in line with decompositional accounts. In real words embedded within naturalistic semantic context, other factors, such as lexical frequency or root frequency, may interact with morphological decomposition. The sensitivity of microsaccades to such interactions remains to be tested in future studies.

Languages vary to a great extent in their morphological structure and in the contribution of morphological information to efficient reading^17^. Hebrew is a language of particular interest in the study of reading due to its rich morphological system^37^ and non-linear (interleaved) structure. Additionally, vowel information is only sparsely represented in Hebrew written text, and is largely inferred from structural word patterns, making rapid structural analysis crucial for decoding Hebrew words. Indeed, morphology plays a central role in Hebrew visual word processing, sometimes abolishing well-established orthographic effects^38–40^. Our findings provide further evidence for automatic morpheme recognition in Hebrew. However, it remains to be seen whether this effect generalizes to languages where morphological information plays a less dominant role in reading and is not necessary for converting print to sound. The classical MIE (exemplified with overt button presses in a lexical decision task) was found to generalize across languages that vary in their morphological richness, such as English, Italian, French, Swedish and Hebrew^18, 19, 41–43^. Future research will determine whether its microsaccadic counterpart generalizes across languages and orthographies.

In addition to the effect induced by root morphemes, our correlation analyses uncovered an association between microsaccade timing and orthographic similarity to real words. Interestingly, this correlation reached significance only for stimuli that did not contain a real root morpheme. A possible interpretation is that when stimuli contain a real root, microsaccade timing may be affected by various properties of the root itself (e.g., the meaning of related words that share the same root). However, in the absence of root semantics, orthographic similarity to real words becomes the most prominent factor influencing processing difficulty, therefore affecting microsaccade timing. This novel finding demonstrates the sensitivity of microsaccades to multiple axes of word knowledge and highlights the potential value of microsaccade-measurements in future psycholinguistic studies of reading, lexical processing and sentence processing.

Several questions remain unanswered by the current study. First, the experimental set-up included presentation of single stimuli in fixed time intervals. It is unclear whether the sensitivity of microsaccades could be observed under more naturalistic reading conditions, such as self-paced reading of words in context. If so, microsaccades can be incorporated in various experiments tapping into language processes beyond the single word level. Second, in this experiment subjects had to make a conscious decision about the stimuli (word or not a word). Future studies would be necessary to assess whether the effects are task-dependent or occur automatically upon perception of orthographic stimuli. Furthermore, as this decision entailed a button press, it is unclear to what extent the effect reflects covert motor preparation. We intentionally avoided motor responses for both pseudoword conditions (as motor responses were shown to affect microsaccade execution)^5^. However, it might be the case that the difference observed in microsaccade timing stemmed from a greater difficulty to inhibit the response to real root pseudowords, compared to invented root pseudowords. In other words, the relationship between microsaccade inhibition and root extraction may be mediated by covert motor inhibition. This possibility remains to be tested in designs that tease apart the motor component from the lexical decision.

Another question concerns the definition of microsaccades. Although many studies use binocularity as a criterion^11, 14, 15, 44, 45^, recent findings suggest that monocular microsaccades can be measured reliably, and might be functionally different than binocular microsaccades^46^. In the current study eye movements were recorded monocularly in order to enhance the quality of data acquisition. Thus, it is currently not possible to tell whether the sensitivity to linguistic information reported here characterizes monocular microsaccades, binocular, or both. Lastly, the baseline rate of microsaccades and blinks showed high inter-individual variability in the current study. In fact, 4 subjects had extremely low blink rate that did not allow reliable analysis. In addition, several subjects showed very early microsaccades, which may have obscured early onset of inhibition effects observed in previous studies of microsaccade inhibition^10, 44^. The current study was not designed to investigate the source of this variability and whether it is unique to this task or reflects oculomotor function in general. Future studies should incorporate a baseline viewing condition and external measures of reading skills in order to tease apart the sources of this variability and whether it has predictive value for reading performance.

It should also be emphasized that microsaccade inhibition may not be directly or functionally linked to language processing, but rather reflects cognitive effort in general. Nevertheless, several factors render microsaccades a promising tool in psycholinguistic research. They occur as early as 300 ms post stimulus and changes in their rate can be reliably measured over time. Compared to response time (RT) in traditional behavioral paradigms, which provides a global measurement of the end result of processing, microsaccades allow inspection of the entire time-course and analysis of intermediate phases of processing as they unfold. The high temporal precision of microsaccades can be used to shed light on the order in which different levels of linguistic information are accessed, a hotly debated topic in the psycholinguistic literature, e.g. refs 29, 47–50. In the current experiment, differences in microsaccade and blink timing were observed even without an overt manual response, suggesting that these novel ocular measures can be used to tap into internal cognitive processes in the absence of subject’s intention or conscious control. In sum, these features of microsaccades may pave the way for studies of language functions in non-communicating or speech impaired individuals, such as people with autism, patients following brain injury, or infants.

## Methods

### Participants

Sixteen undergraduate students from Bar Ilan University participated in the experiment. All participants were native speakers of Hebrew, 18 to 40 years old (mean age 23.6, 3 males), right-handed, with normal or corrected to normal vision and with no reported history of attention or learning disorders or any reported neurological deficit. Participants were rewarded with course credit or payment. The study was conducted in accordance with the Code of Ethics of the World Medical Association (Declaration of Helsinki) and was approved by the local Institutional Review Board of Sheba Medical Center. Written informed consent was obtained from all participants.

### Apparatus

Stimuli were displayed on a 22-inch CRT monitor, running at a 100-Hz refresh rate with 1024 × 768 pixel resolution occupying a 33.4° × 25.4° area. The background luminance was 3.2 cd/m^2^. The experiments were administered in dim light. Eye movements were recorded monocularly with an Eyelink 1000 infrared system (SR Research, Ontario, Canada) with a sampling rate of 500 Hz. Head movements were limited by a chin and forehead rest, placed 60 cm from the screen. Recording was performed from the right eye although viewing was binocular. A standard 9-point calibration was performed before each session. Stimuli were presented using an in-house-developed platform for psychophysics and eye-tracking experiments (PSY) developed by Y.S.B., running on a Windows PC.

### Stimuli and procedure

Hebrew words and pseudowords were presented in a word detection paradigm: Participants were instructed to maintain central fixation and press the left mouse button as quickly and accurately as possible when the letter string presented on the screen was a real Hebrew word. The critical comparison involved 2 pseudoword conditions: Those consisting of a real root and those consisting of an invented root. This allowed us to test for participants’ sensitivity to the morphemic structure of the pseudowords. Word stimuli were included only for the sake of the task (word detection). Stimuli were rendered in Arial font, height: 1.14°–1.6° visual angle, average width: 5.7° visual angle (range 3.2°–9°), at a luminance of 17 cd/m^2^. Stimuli were flashed briefly at the center of fixation (200 ms each, SOA = 1600 ms), while a small (0.13°) dim static circular fixation point was constantly presented. The stimuli and trial sequence are illustrated in Fig. 1. Stimuli were presented in unpointed script, which emulates natural reading conditions for skilled Hebrew readers.

Pseudowords were generated by inserting real or invented Hebrew roots into a fixed set of real patterns, such that the resulting stimulus was always a non-existing root-pattern combination. A total of 240 pseudowords of each type were presented, as well as 480 real words. Thus, the overall proportion of words in the experiment was 50%. Real-root and Invented-root pseudowords were matched on several measures of orthographic similarity to real words (see Table 1). This is critical in this paradigm, because similarity to real words is an important predictor of RT to pseudowords in lexical decision^22, 24, 51^. Pseudowords were screened carefully to exclude homophones and homographs of real Hebrew words using the Hebrew lexicon “Mila”^52^. For further detail on stimulus generation see ref. 20.

The experiment began with ten practice trials that included feedback, followed by 10 blocks of 3 minutes each. Each block began with 2 warm-up trials that were not analyzed, followed by 96 experimental trials- 48 words and 48 pseudowords. Within a block, stimuli were presented in a pseudorandomized order, so that no more than eight words or pseudowords occurred in a sequence, and no semantically related words were presented in consecutive trials.

### Data analysis

We compared measures of microsaccades and blinks in response to *Real*-*root pseudowords* vs. *Invented*-*root pseudowords*. Word-responses are visualized as a descriptive baseline only, but no statistical comparison was conducted between words and pseudowords, because these stimuli are categorically different. Moreover, word-trials involved a motor response, and motion preparation and execution are known to affect oculomotor activity^5^. Data analysis was carried out using in-house software written in Matlab 2013a (The Mathworks, Natick, MA), developed by Y.S.B.

#### Microsaccade and blink detection

Microsaccades were detected using the algorithm introduced by Engbert & Kliegl^11, 53^ adapted for 500 Hz sampling rate as implemented in ref. 10. In the original algorithm, data were recorded binocularly so that temporal overlap between the eyes would serve as an additional criterion for distinguishing microsaccades from noise. Here we chose instead to conduct monocular recordings to improve the spatial resolution of the acquired data. Monocular recordings combined with restrictive thresholds were used successfully in our previous studies^10, 28, 54^.

Raw data were first smoothed using a local linear regression fitting (LOWESS method, span of 25 ms) to optimize microsaccade extraction. Microsaccades were detected as intervals in which the velocity exceeded a threshold defined as eight median standard deviations of the horizontal and vertical velocities (λ = 8). This restrictive threshold (compared with λ = 6 in the original algorithm) was chosen to reduce sensitivity to noise. The minimal microsaccade duration was set to 9 ms. The permitted velocity range was 8–150°/s. Following detection, saccades with amplitude higher than 2° or lower than 0.08° were rejected (see Supplementary Fig. S4 for distribution of microsaccade amplitudes). The rejection rate varied across participants and was in the range of 1–18%, with an average of 8.3%. When analyzing microsaccades, periods of missing data were locally discarded from further analysis with an additional margin of 100 ms, without discarding the whole epoch.

Eye-blinks were detected in three stages. First, blinks were defined as periods of data where the pupil was completely occluded (zero pupil size). Since blinks are typically preceded by an apparent vertical eye movement^55^, the vertical trace was further analyzed in a local window of 100 ms before and 150 ms after each blink, in search for the true onset of the vertical movement preceding the blink. The first third of the local window served to calculate a baseline for transient changes in the vertical trace. The new blink onset was then defined as the time when the change in the vertical trace passed a threshold of 4 standard deviations of that baseline. Blink offset was defined similarly. Lastly, blinks shorter than 250 ms or longer than 700 ms were rejected as possibly reflecting measurement noise. Following extraction, the recorded data were divided into epochs time-locked to stimulus onset, such that each epoch represented one experimental trial.

#### Microsaccade and blink rate modulation

The rate modulation function for both microsaccades and blinks was calculated as in ref. 10, with a small modification. Rates were computed by convolving a raw rate estimate of one microsaccade (or blink) per sample duration at the time of onset with a causal kernel^45^. The rates were averaged across all trials across participants, to compute the event-related modulation of microsaccades (or blinks) with equal contribution from each participant.

#### Microsaccade and blink reaction time

Quantitative measures for the microsaccade- (or blink-) inhibition duration were computed using a method introduced in refs 10 and 21. Microsaccade RT (msRT) was calculated per epoch as the latency of the first microsaccade after stimulus onset, in a time window extending from the stimulus offset (200 ms) to 900 ms post-stimulus. This window was chosen to cover the release from inhibition and exclude irrelevant late effects (see Supplementary Figs S5–6 for parallel analyses with alternate time windows). Blink RT (bkRT) was similarly calculated, as the latency of the first blink after stimulus onset in the same time window. Epochs with no microsaccades or blinks in the specified window were not included in this calculation. In computing error bars for the RT values averaged across subjects, we applied the Cousineau method, which controls the between-subject variance and allows a better representation of within-subject effects^56^. In this method, data are first normalized by subtracting each subject’s mean RT and adding the group mean RT across all conditions and subjects. The standard error is calculated over the normalized data. We then multiply the error bars by Morey’s correction factor^57^ (√(n/(n − 1)), where n is the number of conditions; in our case this factor equals √(3/2)).

We used nonparametric permutation tests^58^ to test the difference in msRT between the Real-root and Invented-root conditions. For each test, we randomly permuted the labels of the epochs (1,000 permutations) and recalculated group average msRT. We then computed the p value as the fraction of permutations in which the original effect size (i.e., the difference between the conditions divided by the pooled standard deviation) was exceeded by the effect size of the permuted data. The same procedure was used to assess the significance of the differences between in bkRT for the Real-root and Invented-root conditions.

#### Association between msRT and word similarity

For each pseudoword condition, we calculated Pearson’s correlation coefficients between the msRT to each stimulus and known measures of orthographic word similarity: (a) Coltheart’s N, the number of real word neighbors^24^. (b) OLD20, the mean orthographic Levenshtein distance to the 20 closest orthographic neighbors. OLD is defined as the number of insertions, deletions and substitutions needed to generate one letter string from another. OLD20 is the average of this distance across the 20 closest real word neighbors^23^. (c) Mean Log Bigram Frequency (MLBF), a measure of sublexical orthographic familiarity. The position specific bigram frequency of a given bigram is the frequency with which that bigram occurs at the same sequential position among all words of the same length. MLBF averages the log value of this positional bigram frequency for all bigrams in a given string. For further details on calculating these measures see ref. 20. To discard individual variability in overall msRT, we first subtracted each participant’s average msRT from the msRT obtained for each stimulus (∆msRT), then averaged the result across participants, per stimulus.

Significance was controlled for false discovery rate (FDR) across the 6 comparisons (2 conditions × 3 word-similarity measures) at a 5% criterion, and 95% confidence intervals (CI) were calculated using a bootstrap procedure with 1,000 iterations^58^. Lastly, we used Fisher’s r-to-z transformation to determine the significance of the difference between the correlation coefficients in the two conditions.

## Electronic supplementary material

Supplementary Figures S1-S6
