# Supplementary material for: Microsaccades are sensitive to word structure: A novel approach to study language processing
Source: Sci Rep. 2017 Jun 21;7:3999. doi: 10.1038/s41598-017-04391-4 (PMC5479819; doi:10.1038/s41598-017-04391-4)
Supplement: Supplementary file 1 — Supplementary Figures S1-S6 [file 41598_2017_4391_MOESM1_ESM.pdf]

Microsaccades are sensitive to word structure: A novel approach to study language processing

Maya Yablonski[1*], Uri Polat[2], Yoram S. Bonneh[2§] and Michal Ben-Shachar[1, 3§]

[1]The Gonda Multidisciplinary Brain Research Center, Bar Ilan University, Ramat-Gan, Israel

[2]School of Optometry and Vision Science, Mina & Everard Goodman Faculty of Life Sciences, Bar Ilan University, Ramat-Gan, Israel

[3]Department of English Literature and Linguistics, Bar Ilan University, Ramat-Gan, Israel

[§]These authors jointly supervised this work.

[*]Corresponding author, mayayab@gmail.com

Supplementary Figure S1

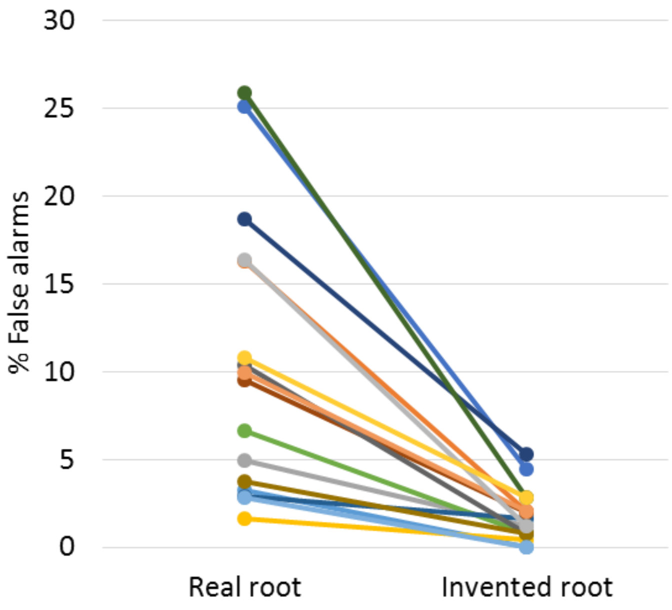

Individual performance in the word detection task for each subject (N = 16). False alarm ratios (the likelihood of issuing a "Word" response to a pseudoword stimulus) were significantly higher for Real-root pseudowords compared to Invented-root pseudowords ($t(15) = 5.19$; $p < 0.001$).

Supplementary Figure S2

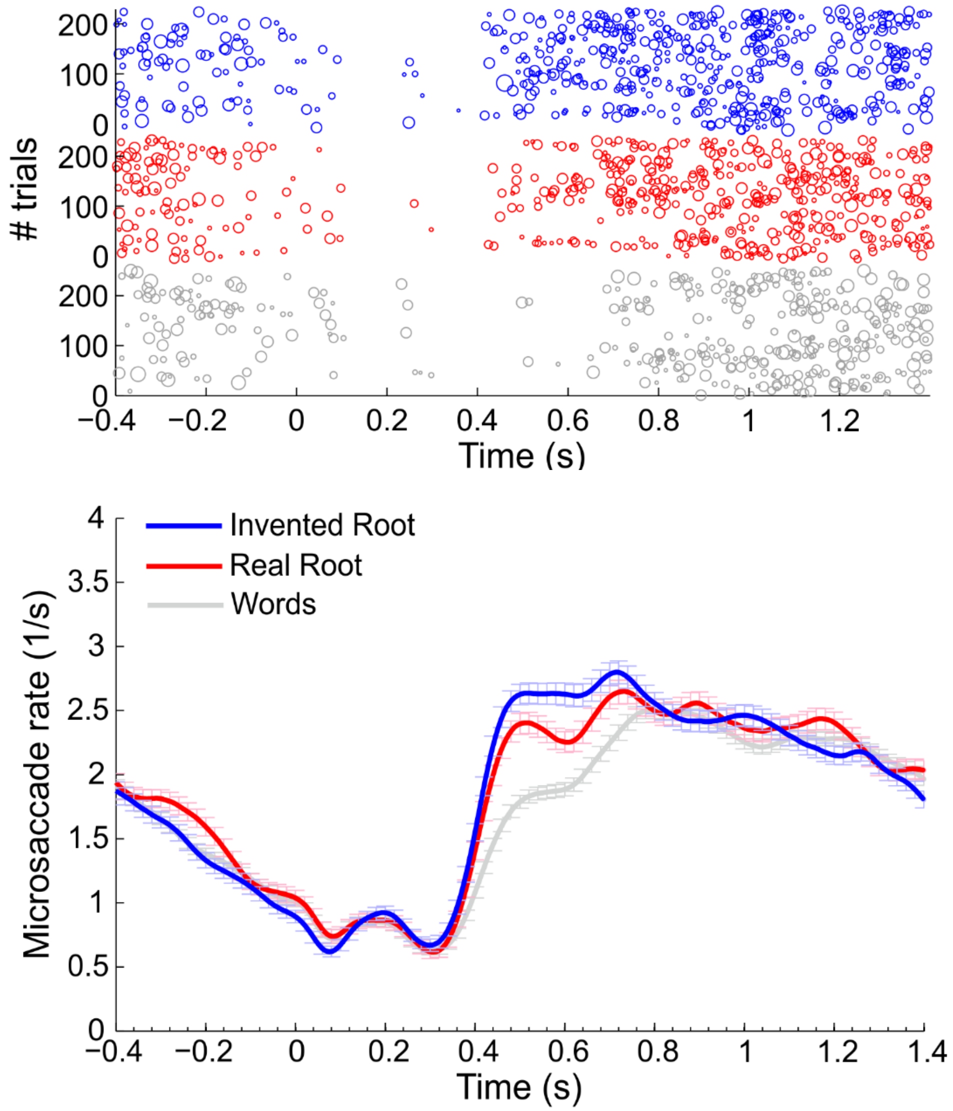

Microsaccade rate modulation. This figure parallels Figure 2 in the main manuscript after setting the upper magnitude threshold to 1°. (a) Raster plots of microsaccades in a single subject (female, 24y) for Invented-root pseudowords (blue), Real-root pseudowords (red) and Words (gray). Each row represents a single trial. Each circle represents a single microsaccade, with circle diameter proportional to microsaccade magnitude. For visualization, all pseudoword trials (N=240 trials per condition) but only half of the word trials (N=240) are displayed. (b) Average microsaccade rate modulation curves. Data were averaged, per condition, across all trials and all subjects. Error bars denote standard error of the mean across all trials in each condition. Time zero represents stimulus onset.

Supplementary Figure S3

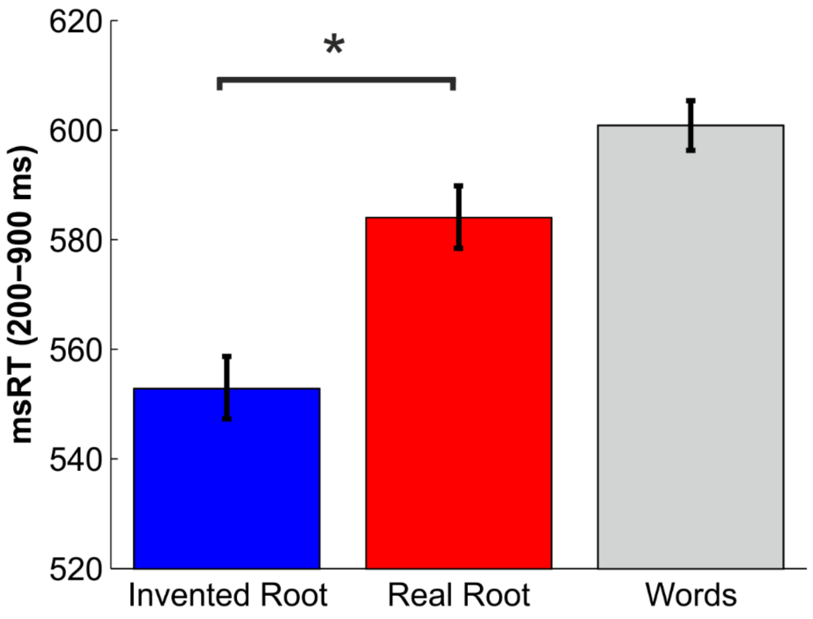

The effect of morphological structure on microsaccade inhibition. This figure parallels Figure 3 in the main manuscript after setting the upper magnitude threshold to 1°. msRT is presented for each condition (same color scheme as in Fig. 2). Only trials that included a microsaccade in the specified time window (200-900ms) were included in the calculation (about 60% of the trials in each condition). Data were normalized (de-meaned) per subject, averaged across subjects (N=16) and adjusted by adding the group grand-average (see Methods). Error bars denote standard errors across subjects. Microsaccades in Real-root trials were significantly delayed compared to Invented-root trials (nonparametric permutation test). $*p < 0.001$.

Supplementary Figure S4

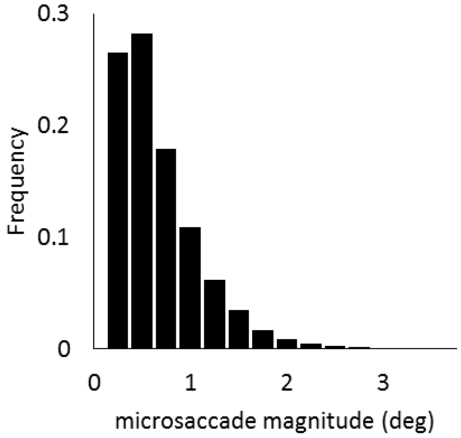

Distribution of microsaccade magnitudes for all subjects (N = 16, total number of microsaccades = 59993). Across subjects, 84% of microsaccades were smaller than 1°. This is in accordance with previous reports, e.g. supplementary ref [1].

Supplementary Figure S5

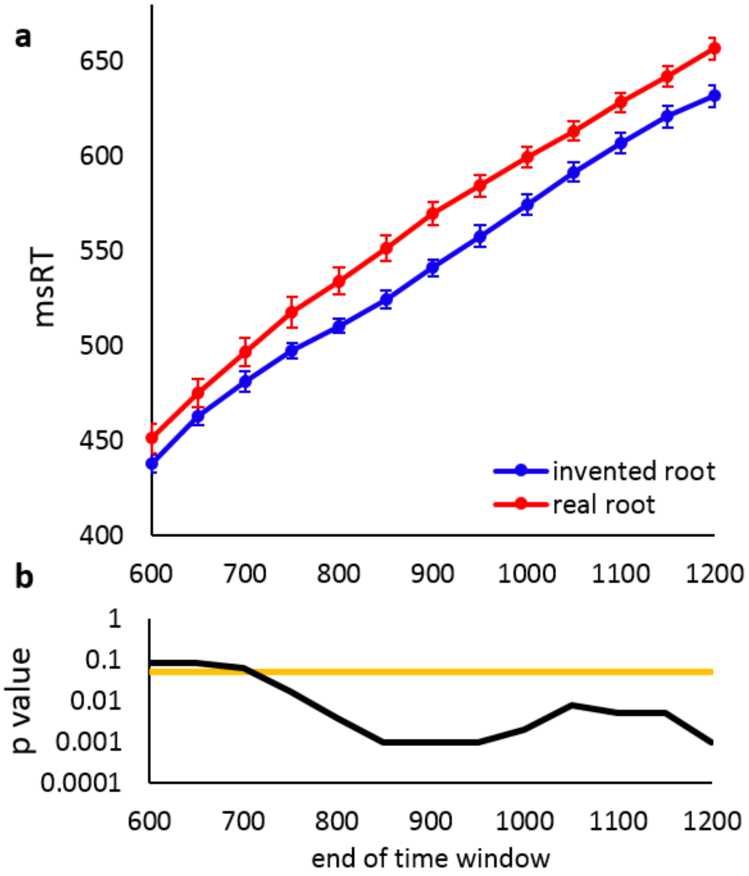

msRT as a function of window size. (a) Average msRT for invented root pseudowords (blue) and real root pseudowords (red). Error bars denote standard errors across subjects (N=16). (b) The level of significance of the morpheme interference effect evaluated using a non-parametric permutation test (1,000 permutations, black line). P values on the y-axis are presented on a logarithmic scale. The orange line depicts the significance threshold of 0.05. This analysis demonstrates that the MIE in microsaccades is significant across a range of time windows (200:750 through 200:1200).

Supplementary Figure S6

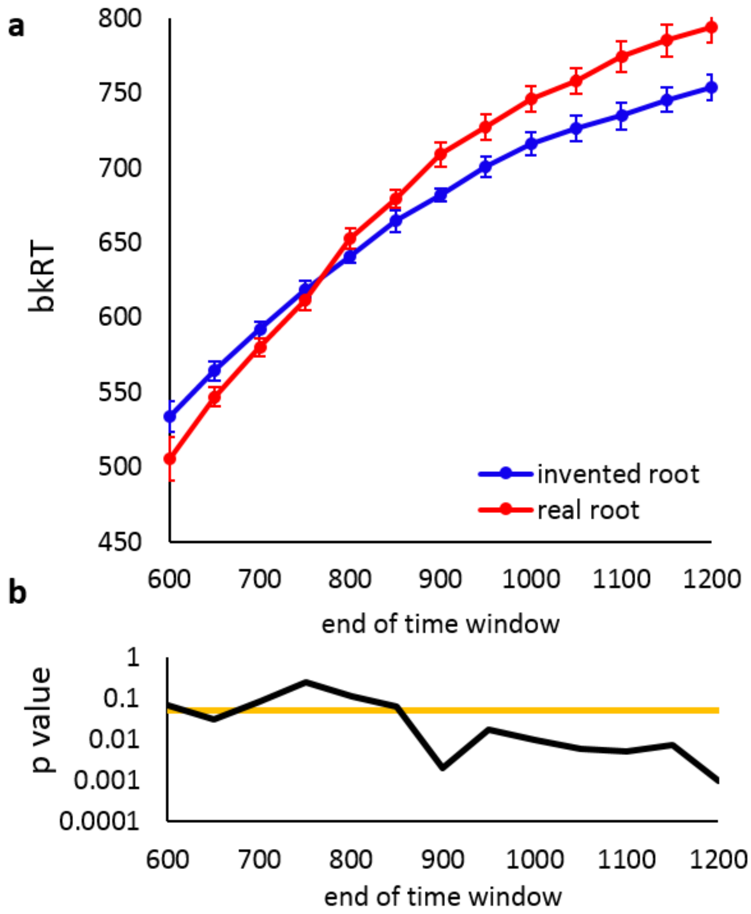

bkRT as a function of window size. (a) Average bkRT for invented root pseudowords (blue) and real root pseudowords (red). Error bars denote standard errors across subjects (N=12). (b) The level of significance of the morpheme interference effect evaluated using a non-parametric permutation test (1,000 permutations, black line). P values on the y-axis are presented on a logarithmic scale. The orange line depicts the significance threshold of 0.05. This analysis demonstrates that the MIE in blinks is significant across a range of time windows (200:850 through 200:1200).

References

1. Otero-Millan, J., Macknik, S. L. & Martinez-Conde, S. Microsaccades and Blinks Trigger Illusory Rotation in the 'Rotating Snakes' Illusion. *J. Neurosci.* **32,** 6043–6051 (2012).
